# The mitochondrial ribosomal protein of the large subunit, Afo1p, determines cellular longevity through mitochondrial back-signaling via TOR1

**DOI:** 10.18632/aging.100065

**Published:** 2009-07-13

**Authors:** Gino Heeren, Mark Rinnerthaler, Peter Laun, Phyllis von Seyerl, Sonja Kössler, Harald Klinger, Stefanie Jarolim, Birgit Simon-Nobbe, Matthias Hager, Christoph Schüller, Didac Carmona-Gutierrez, Lore Breitenbach-Koller, Christoph Mück, Pidder Jansen-Dürr, Alfredo Criollo, Guido Kroemer, Frank Madeo, Michael Breitenbach

**Affiliations:** ^1^Department of Cell Biology, Division of Genetics, University of Salzburg, 5020 Salzburg, Austria; ^2^Department of Biochemistry and Cell Biology, Max F Perutz Laboratories, University of Vienna, 1030 Vienna, Austria; ^3^Institute of Molecular Biosciences (IMB), University of Graz, 8010 Graz, Austria; ^4^Molecular and Cell Biology Division, Institute for Biomedical Aging Research of the Austrian Academy of Sciences, 6020 Innsbruck, Austria; ^5^INSERM, U848, Institut Gustave Roussy, PR1, 39 rue Camille Desmoulins, F-94805 Villejuif, France

**Keywords:** Saccharomyces cerevisiae, yeast mother cell-specific ageing, TOR complex, rapamycin

## Abstract

Yeast mother cell-specific aging constitutes a model of replicative aging as it occurs in stem cell populations of higher eukaryotes. Here, we present a new long-lived yeast deletion mutation,afo1 (for aging factor one), that confers a 60% increase in replicative lifespan. AFO1/MRPL25 codes for a protein that is contained in the large subunit of the mitochondrial ribosome. Double mutant experiments indicate that the longevity-increasing action of the afo1 mutation is independent of mitochondrial translation, yet involves the cytoplasmic Tor1p as well as the growth-controlling transcription factor Sfp1p. In their final cell cycle, the long-lived mutant cells do show the phenotypes of yeast apoptosis indicating that the longevity of the mutant is not caused by an inability to undergo programmed cell death. Furthermore, the afo1 mutation displays high resistance against oxidants. Despite the respiratory deficiency the mutant has paradoxical increase in growth rate compared to generic petite mutants. A comparison of the single and double mutant strains for afo1 and fob1 shows that the longevity phenotype of afo1 is independent of the formation of ERCs (ribosomal DNA minicircles). AFO1/MRPL25 function establishes a new connection between mitochondria, metabolism and aging.

## Introduction

Yeast (*Saccharomyces cerevisiae*) mother cell-specific aging has been shown to be based on the asymmetric distribution of damaged cellular material including oxidized proteins [1]. The mother cell progressively accumulates this material and ages depending on the number of cell division cycles, while the daughter "rejuvenates" and enjoys a full lifespan. Young daughter cells and old (senescent) mother cells can be efficiently separated based on their different size, by elutriation centrifugation [2].

At least some biochemical and genetic mechanisms of aging are conserved throughout the evolution of eukaryotes. A prominent hypothesis postulates that the progressive deterioration of mitochondrial metabolism leads to the production of reactive oxygen species (ROS) that oxidize vulnerable cellular proteins and lipids, while damaging the genome. The cell's genetic response to this oxidative stress may appear as a "genetic program of aging". In this light, some of the current aging theories could well be interrelated and compatible among each other (for review, see [3,4]).

The TOR signaling pathway is highly conserved from yeast to human cells [5]. It regulates nutrient responses by modulating the nucleo-cytoplasmic shuttling of transcription factors including Sfp1p, which governs ribosome biosynthesis [6]. Down-regulation of TOR kinase induces entry into stationary phase and stimulates autophagy, a process that is vital for survival in conditions of starvation [7]. TOR kinase activity may also be involved in the retrograde response of cells that adapt their nuclear transcriptome to defects in mitochondrial respiration [8]. Yeast possesses two closely related proteins, Tor1p and Tor2p, forming two "TOR complexes" among which only one, TORC1 (containing either Tor1p or Tor2p and active in growth control), is inhibited by rapamycin. Deletion of *TOR2 *is lethal due to its essential function in TORC2 (acting on determination of cell polarity). Deletion of *TOR1 *leads to an increase in mitochondrial respiration and protein density [9,10] and to a 15% increase in replicative lifespan, thus establishing a link between nutrition, metabolism, and longevity [11].

In this paper we are presenting a novel long-lived mutant of yeast that establishes a new connection between mitochondria, metabolism and aging. The life-prolonging mutation affects a gene encoding a mitochondrial ribosomal protein, leads to respiratory deficiency, and relies on *TOR1 *to confer longevity.

## Results

### A novel yeast mutant with reduced replicative aging

**Figure 1.**
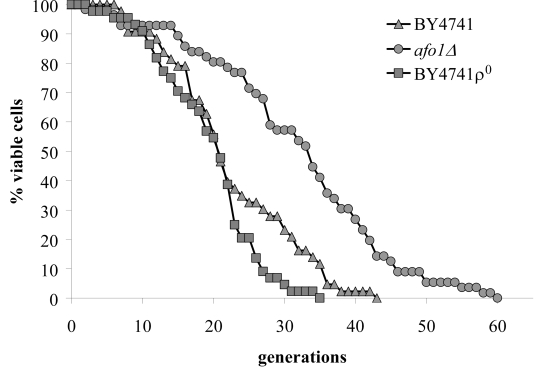
Lifespans of isogenic strains *afo1Δ*, wild type BY4741 and BY4741 ρ^0^. Lifespans were determined as described previously [2] by micromanipulating daughter cells and counting generations of at least 45 yeast mother cells on synthetic complete (SC) media with 2% glucose as carbon source.

We compared the transcriptome of senescent yeast mother cells (fraction V) with young daughter cells (fraction II) after separating them by elutriation centrifugation [2]. Senescent cells were found to upregulate 39 genes and to down-regulate 53 transcripts. Deletion mutants [12] corresponding to these 92 genes were tested for their resistance or hypersensitivity to five different oxidants (hydrogen peroxide, *tert*-butyl hydroperoxide (t-BHP), diamide, cumene hydroperoxide, and menadione). Only two mutants were found to be consistently resistant against more than one oxidant (and not hypersensitive to any other oxidant). Among these two mutants only one, deleted for YGR076C/*MRPL25 *(later termed *AFO1*, see below) caused a mother cell-specific lifespan expansion on the standard media used by us (SC + 2% glucose) (Figure 1). This deletion mutation conferred resistance to diamide and t-BHP and a somewhat weaker resistance to hydrogen peroxide, as well as a 50% reduced ROS production (as compared to the BY4741 ρ^0^mutant). ROS production was measured by quantitation of fluorescence signals obtained after dihydroethidium (DHE) staining. The mutant displayed a 60% increase in the median and a 71% increase in the maximum lifespan (Figure 1). The mutant only grew on media containing fermentable carbon sources and hence is respiration deficient. We therefore asked if the respiratory deficiency caused the increased replicative life span. However, a *bona fide *BY4741 ρ^0^mutant did not show any extension in replicative life span (as compared to BY4741 WT cells), meaning that lack of respiration is not sufficient to confer longevity to mother cells (Figure 1). We also tested if *afo1*Δ cells displayed the retrograde response [3,13] by measuring *CIT2 *transcription and no effect of the *afo1*Δ mutation could be discerned (see Supplementary Figure 1). We conclude that the elongation of lifespan observed here is not caused by respiratory deficiency and is independent of the retrograde response as defined by Jazwinski [14] and Butow [15].

### *AFO1 *codes for a mitochondrial ribosomal protein

*AFO1 *(YGR076C) codes for MrpL25p, identified by proteomic analysis as a component of the large subunit of the mitochondrial ribosome [16]. Because of its remarkable longevity phenotype, we re-named the gene *AFO1*(for aging factor one). A recombinant construct in which Afo1p was fused in its C-terminus with GFP (Afo1-GFP) was transfected into a heterozygous *afo1Δ*strain. Tetrad dissection revealed that the Afo1-GFP could replace endogenous Afo1p to enable growth on a non-fermentable carbon source. Confocal fluorescence microscopy confirmed that the protein is located in mitochondria irrespective of the cellular age and the genetic background (supplementary material, Supplementary Figure 2). The deletion mutant *afo1Δ *exhibited a ρ^0^petite phenotype, meaning that it failed to grow on glycerol media and lacked DAPI-detectable mitochondrial DNA. The mutant also showed negligible oxygen consumption when growing on glucose (data not shown). However, in contrast to a *bona fide *BY4741 ρ^0^petite mutant, which grew much more slowly than WT cells on standard media with 2% glucose as carbon source, the *afo1*Δ mutant grew as rapidly as WT cells. The growth properties of the mutant and its metabolic implications will be published in detail elsewhere. The average size of the *afo1*Δ mutant cells in exponential phase was equal to that of WT cells, while cells of the ρ^0^strain were about 20% larger. Importantly, disruption of the *AFO1 *gene in ρ^0^cells restored rapid growth, hence reversing the growth defect induced by the absence of mitochondrial DNA.

To obtain definite genetic proof that the *AFO1 *deletion caused the resistance against oxidative stress and the extension of the life span described above, we performed co-segregation tests in meiotic tetrads after out-crossing the *afo1*Δ strain in an isogenic cross.  In 10 unselected tetrads, which all revealed a regular 2:2 segregation, we observed strict co-segregation of G418 resistance indicating the presence of the gene deletion, respiratory deficiency, and resistance against hydrogen peroxide stress (Figure 2A). We also tested the lifespan of all four haploid progeny of one tetrad and found consistent co-segregation of the deletion allele of *afo1 *with extended lifespan (Figure 2B). Furthermore, we also tested if the phenotype of the mutant might result from changes of the expression of the two neighboring genes of *AFO1*. No such effect was apparent (Figure 2C). We conclude that lack of *AFO1 *results in long lifespan and the oxidative stress resistance.

**Figure 2A.**
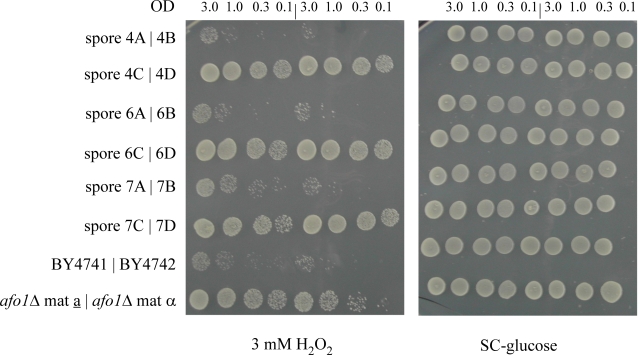
Segregation of the mutant phenotypes of *afo1Δ* in meiotic tetrads after outcrossing and influence of the genes adjacent to *AFO1*. 10μl aliquots of the cultured strains were spotted on SC-glucose and on SC-glucose + oxidants, as indicated in the figure. Cultures were grown to OD_600_ = 3.0 and diluted as indicated. Three out of ten tetrads tested are shown together with two wild type and two *afo1* deletion strains.

**Figure 2B.**
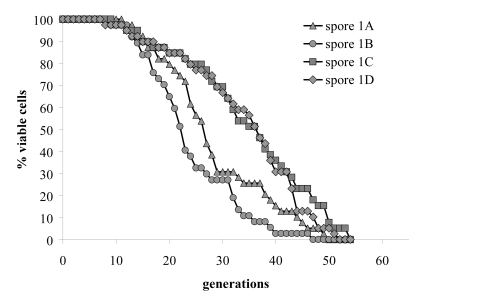
Replicative lifespans of the four haploid segregants of one meiotic tetrad were determined.

**Figure 2C.**
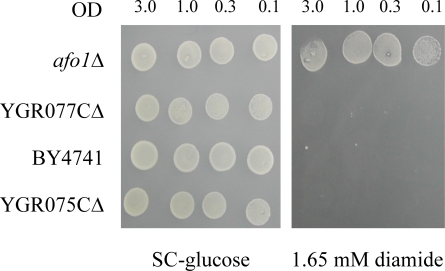
Deletion strains corresponding to the two genes adjacent to *AFO1* are shown. These deletions have no influence on the resistance to oxidants.

**Figure 3.**
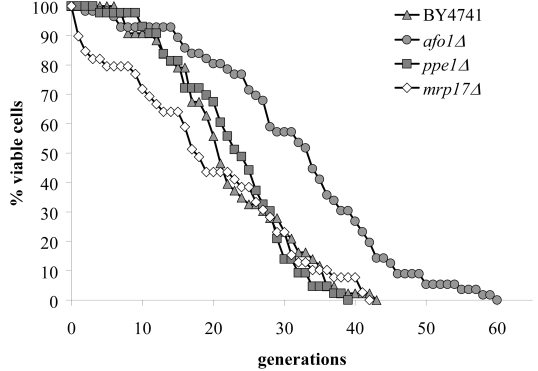
Lifespans of the strains deleted for *ppe1 *and *mrp17. *The single deletion strains for YKL003C (encoding for Mrp17p) and YHR075C (encoding for Ppe1p), both of the mitochondrial ribosomal small subunit, were tested for their lifespan. The strains were constructed in the BY4741 background. The measured lifespans were not significantly different from wild type (p<0.02).

We next addressed the possibility that the deletion of other mitochondrial ribosomal genes might also lead to an increase in replicative life span. For this, we investigated the lifespan, growth properties and oxidative stress resistance of two additional deletion mutants in the genes *MRP17 *and *PPE1*, encoding mitochondrial ribosomal proteins of yeast. YKL003CΔ (*mrp17Δ*) was found to be resistant against diamide, t-BHP and juglone, but was hypersensitive to hydrogen peroxide and had a normal lifespan. YHR075CΔ (*ppe1Δ*) was resistant against diamide, yet had a normal lifespan (Figure 3). Therefore, the effect of the *afo1*Δ deletion mutant on lifespan is gene-specific.

### Longevity mediated by the *afo1 *deletion is mediated by the *TOR1 *pathway

Two independent lines of evidence revealed that the *afo1 *deletion confers longevity and oxidative stress resistance through the *TOR1 *signaling pathway. First, we chromosomally integrated a C-terminally GFP-labeled version of the transcription factor, Sfp1p, at the *SFP1 *locus under the control of the native promotor in strains *afo1Δ, *BY4741 WT and BY4741 ρ^0^. Sfp1p is activated by the TOR1 and PKA pathways and is regulated by shuttling between the nucleus in its active form and the cytoplasm upon deactivation. Sfp1p is a major regulator of cytoplasmic ribosome synthesis and, consequently, of cellular growth [6]. As expected, addition of the Tor1p inhibitor rapamycin to WT cells induced the translocation of Sfp1p from the nucleus to the cytoplasm. In the *bona fide *BY4741 ρ^0^strain, Sfp1p was found constitutively in the cytoplasm, even in the absence of rapamycin. In stark contrast, in the *afo1*Δ mutant, Sfp1p was constitutively present in the nucleus, and rapamycin failed to induce the nucleo-cytoplasmic translocation of Sfp1p (Figure 4A). Similar results were obtained with an alternative Tor1p inhibitor, arsenite [17].  Arsenite induced the nucleocyto-plasmic translocation of Sfp1p in WT cells, while Sfp1p stayed in the cytoplasm of ρ^0^cells and in the nuclei of *afo1 *mutant cells, irrespective of the addition of arsenite (Figure 4B). Rapamycin failed to inhibit the growth of *afo1 *mutant cells [18]. Altogether, these data suggested that *TOR1 *signaling might govern the longevity of *afo1 *cells. The relation between *TOR1 *and *AFO1 *was further explored by epistasis experiments using double mutants (Figure 5). The lifespan of the double deletion strain (*afo1*Δ, *tor1Δ*) was similar to the lifespan of the *tor1 *deletion strain, i.e. about 15% longer than wild type (in good agreement with [11]). However, the double mutant *afo1*Δ,*tor1Δ *strain aged more rapidly than the single mutant *afo1*Δ strain (Figure 5). We conclude that a functional *TOR1 *gene is needed for exerting the lifespan-prolonging effect of *afo1*Δ.

We constructed single and double knockout *afo1*Δ, *sfp1*Δ mutant strains and tested their mother cell-specific lifespan and oxidative stress resistance. The median lifespan of *sfp1*Δ cells was shortened considerably as compared to WT cells, and the lifespan of the double *afo1*Δ, *sfp1*Δ mutant was longer than that of the *sfp*1Δ mutant, yet shorter than WT and *afo1*Δ (Figure 6A). Hence, the very short lifespan of *sfp1*Δ mutant cells is partially rescued by the *afo1 *mutation. Like the double *afo1*Δ,*sfp1*Δ mutant, *sfp1Δ *cells displayed a major growth defect. When the *sfp1*Δ strain was made ρ^0^with ethidium bromide, cell growth was not further inhibited (data not shown). Comparison of the strains on plates containing 1.6 mM t-BHP  revealed that *afo1Δ *is moderately resistant, while single *sfp1Δ *and double *afo1*Δ,*sfp1*Δ mutants exhibited a similar degree of high resistance (Figure 6B). Taken together, these results show that the lifespan-extending effect of *afo1Δ*is most likely independent of the presence of *SFP1*.

**Figure 4A.**
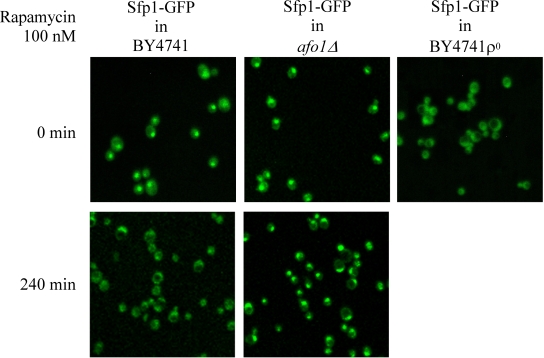
Influence of rapamycin on subcellular localization of the transcription factor, Sfp1p. Strains were grown in liquid SC+2% glucose at 28°C until early logarithmic phase and rapamycin was added to a final concentration of 100 nM. This concentration is growth inhibitory for the wild type strain [6]. Confocal images were taken at time zero (before addition of rapamycin) and at 4 h. The chromosomally integrated *SFP1-GFP-HIS3* construct [37] was present in the wild type strain BY4741, was PCR cloned, sequenced and chromosomally integrated at the SFP1 locus in strains *afo1Δ* and BY4741 ρ°, respectively.

**Figure 4B.**
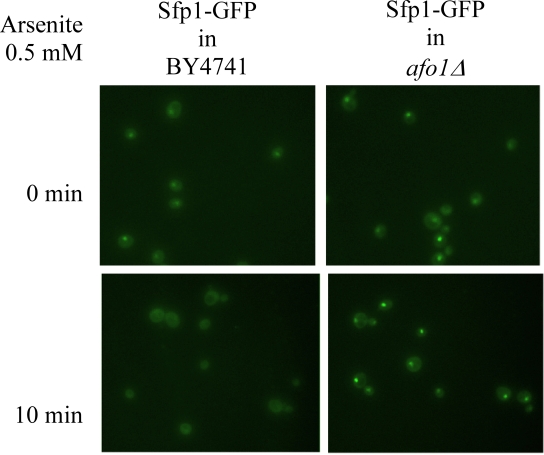
The same strains as in **A** were treated with 0.5 mM arsenite for 10 min.

**Figure 5.**
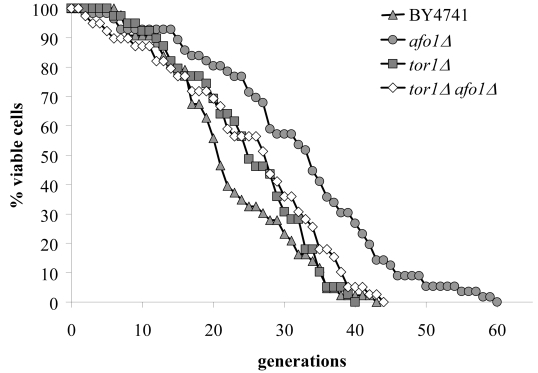
Double mutant experiments of *afo1Δ *and *tor1Δ*. The *TOR1 *gene is involved in nutrient sensing and lifespan determination in yeast [5]. The double mutant was constructed in an isogenic cross between the two single mutants in the BY background. Lifespans of the wild type, both single mutants and the double mutant were determined by micromanipulation. The experiment shows that an intact *TOR1 *gene is needed for the lifespan elongation observed in the *afo1Δ *strain as the lifespan of the *afo1Δ, tor1Δ *double mutant strain is not significantly different (p<0.02) from the lifespan of the *tor1Δ *single mutant strain.

Next, we addressed the question as to whether the longevity phenotype of the *afo1 *mutation might originate from suppressing the yeast apoptosis pathway. As shown previously [2], old mother cells of the wild type display all of the known markers of yeast apoptosis while these markers are absent from young cells. To tackle this problem, we isolated young (fraction II) and old cells (fraction V) from WT and *afo1*Δ cells by elutriation centrifugation and tested several markers of apoptosis such as externalization of phosphatidyl serine and DNA strand breaks (Figure 7). Our data clearly indicated that *afo1*Δ cells did not lose the ability to undergo apoptosis. In spite of a 60% longer median lifespan, senescent mother cells finally succumbed to apoptosis. We conclude that the components of the programmed cell death pathway that a yeast cell has at its disposal, do not cause replicative aging, but that *vice versa *replicative aging finally leads to cell death via apoptosis.

We also investigated whether the longevity phenotype of the *afo1Δ *mutant might be mechanistically related to the production of extrachromosomal rDNA minicircles (ERCs) [19,20]. *FOB1 *encodes a protein required for the unidirectional replication fork block in rDNA replication. We analyzed the influence of the *fob1 *mutation on longevity, growth, and ERC content of WT and *afo1Δ *cells. In our analysis the *fob1Δ *mutation in the BY4741 strain leads to an increase of the replicative lifespan by about 5 generations, in good agreement with previous reports [11,21].

However, we observed a similar median life span of the *fob1Δ, afo1*Δ double mutant and the *afo1*Δ mutant cells (Figure 8A). As an internal control, both the *fob1*Δ single mutant and the *fob1Δ, afo1*Δ double mutant exhibited the absence of ERCs even in fraction IV and V old cells, while a continuous age-dependent increase in ERCs was found, in particular in fraction IV and V senescent mother cells from WT and *afo1*Δ cells (Figure 8B). Thus, the lifespan-extension observed in the *afo1*Δ strain occurs in the presence of ERCs and is not further increased when ERCs are absent, consequently ERCs do not influence longevity in the *afo1Δ *strain.

**Figure 6A.**
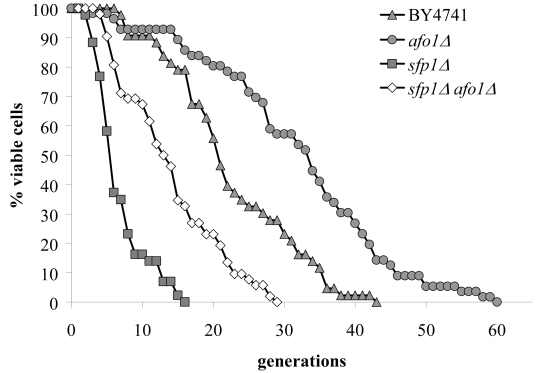
The double mutant strain, *sfp1Δ*, *afo1Δ* was constructed as described in the Materials and Methods section, tested for lifespan, and compared with both single mutant strains and the wild type. The sfp1? strain grows very slowly although it is respiratory-competent, is highly resistant to t-BHP and is very short-lived. The short lifespan of *sfp1Δ* is partially rescued by *afo1Δ*.

**Figure 6B.**
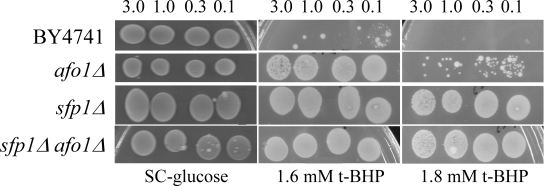
The same strains as in **A** were tested for resistance against oxidative stress induced by 1.6 mM and 1.8 mM t-BHP. The strong resistance of the *sfp1Δ* mutant strain is not rescued by the *afo1* mutation.

## Discussion

### *AFO1*, the retrograde response and mitochondrial back-signaling

The retrograde response (as defined by Jazwinski [14] and Butow [15]) of non-respiring cells is transmitted through the transcription factor Rtg1/Rtg3p and allows for the transactivation of genes involved in peroxisome synthesis that compensate for the deficient amino acid biosynthesis of cells that lack a complete citrate cycle. As an indicator of the retrograde response, expression of the peroxisomal Cit2p citrate synthase is usually measured [14,15]. Yeast strains displaying a strong retrograde response increase their replicative lifespan as ρ^0^strains over that of the corresponding ρ^+^strain. The retrograde response is generally suppressed in 2% glucose but strong on raffinose as sole carbon source [13]. We have measured *CIT2 *transcription under the conditions used in this study and found no increase in the transcript of this gene (Supplementary Figure 1), explaining why the ρ^0^strain in the BY4741 series shows the same lifespan as wild type. When raffinose was used as a carbon source, *CIT2 *transcription was increased, and as expected, an increase in the lifespan of the *bona fide *BY4741 ρ^0^strain was observed (unpublished observation). However, during growth on 2% glucose when the retrograde response is absent in our strain background, we do observe the increase in lifespan described in the present paper. We therefore conclude that the mechanism leading to this increase must be different from the retrograde response.

The lifespan elongation described for the *afo1*Δ mutant strain depends on a signal transmitted from mitochondria to the nucleo-cytoplasmic protein synthesis system and has a strong influence on replicative aging, vegetative growth, and oxidative stress resistance (see below). We propose to call this regulatory signaling interaction „mitochondrial back-signaling" to dis-tinguish it from the retrograde response described by Jazwinski [14] and Butow [15].

### Evidence for involvement of the TOR1 pathway in longevity of the *afo1 *deletion strain

The nature of the signal created by Afo1p is unknown, especially since we found this ribosomal protein to be located in mitochondria in all physiological situations tested, including senescent yeast mother cells. Nonetheless, two independent lines of evidence support the notion that increased activity of *TOR1 *determines the longevity of the *afo1 *deletion mutant. First, in the double mutant deleted for both *TOR1 *and *AFO1*, a lifespan is observed that is only moderately longer than that of the wild type and is identical with the lifespan of the *tor1*Δ single mutant (Figure 5). Thus, paradoxically, the relatively small but significant elongation of the lifespan of ρ^+^respiring *tor1*Δ cells depends on inactivation of Tor1p, while the large increase in lifespan in the non-respiring *afo1Δ *cells depends on activity of the Tor1p. Second, rapamycin fails to abolish the nuclear location of the transcription factor, Sfp1p, an indicator of Tor1p activity, in *afo1Δ *cells (Figure 4A). Likewise, arsenite, another inhibitor of Tor1p [17], fails to abolish the nuclear location of the transcription factor, Sfp1p, in *afo1*Δ cells (Figure 4B). Sfp1p is well known to be one of the major metabolic regulators of growth and ribosome biosynthesis, which is limiting for growth [6]. The data presented here seem to indicate that Sfp1p activity in the nucleus could be crucial for longevity. The double mutant experiments shown in Figure 5 indicate that the *sfp1*Δ and the *afo1*Δ mutations exert their influence on longevity independently of each other. Moreover, we tested TORC1 kinase activity in WT, ρ^0^and in *afo*1Δ cells (data not shown) and found that the long-lived mutant, like the ρ^0^strain displayed only very weak TORC1 kinase activity. These results indicate that the Tor1p activity needed for longevity in the mutant might be feedback-regulated by Sfp1p and/or maybe  independent of TORC1kinase activity.

**Figure 7.**
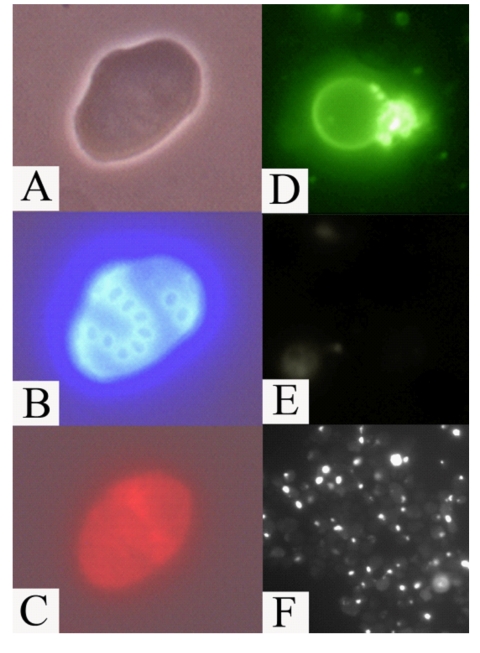
Apoptotic markers in old mother cells (fraction V) of the mutant *afo1Δ *strain. (**A**) phase contrast; (**B**) same cell as in A stained with Calcofluor White M2R; (**C**) the same cell stained with DHE indicating a high level of ROS; (**D**) an old mother cell stained with FITC-annexin V revealing inversion of the plasma membrane; (**E**) the same cell as in (**D**) shows absence of staining with propidium iodide revealing intact plasma membrane; (**F**) TUNEL staining of old *afo1Δ *cells.

A tentative scheme describing the genetic interactions of mitochondrial back-signaling that we are discussing here, is presented in Figure 9.

**Figure 8A.**
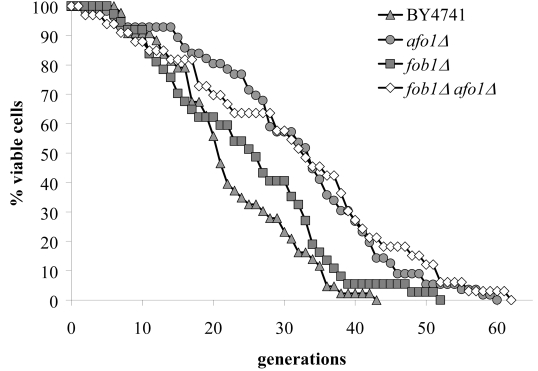
The two mutations, *fob1Δ* and *afo1Δ* were combined in a haploid strain from a meiotic tetrad obtained from an isogenic cross. Wild type, the two single mutants and the double mutant were tested for mother cell-specific lifespan. The fob1 mutation does not further increase the lifespan of the *afo1* mutant strain (p<0.02).

**Figure 8B.**
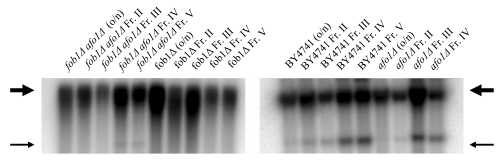
Old and young cells of the same strains as in **A** were isolated by elutriation centrifugation and ERCs were analyzed by gel electrophoresis and Southern blotting with an rDNA-specific probe as described in [19]. Thick arrow: chromosomal rDNA repeats; Thin arrow: ERCs (minicircles). Taken together, the results presented in this figure indicate that longevity in the *afo1Δ* strain is not influenced by the fob1-deletion or the presence of ERCs.

**Has the function of *AFO1 *been conserved in evolution**?

In this paper we are presenting evidence for a possibly indirect interaction of the mitochondrial ribosomal protein, Afo1p, with the *TOR1 *signaling system of yeast. This interaction is independent of the primary function of Afo1p in translation. A reduction of *TOR1 *signaling in yeast [7], rodent and human cells [22] suppresses cellular aging in cell culture [22], and increases longevity in mice [23], worms [24] and fruit flies [25,26]. These effects were shown to be non-additive with caloric restriction suggesting that the TOR pathway in these organisms is crucial for transmitting the caloric restriction signal. In metazoa, cellular life, **but not organismic life **is possible in the absence of mitochondrial respiration. It is therefore difficult to draw conclusions as to the generality of the *afo1 *mutant-based longevity described in the present paper.

The protein complement of mitochondrial ribosomes of both yeast and human cells has been studied [16 and the literature cited therein, 27-30] and the non-translational or extra-ribosomal functions (mostly in transcriptional regulation) of ribosomal proteins have been extensively studied [31,32]. The published extraribosomal functions mostly concern cytoplasmic, not mito-chondrial ribosomes. Possible extraribosomal functions have to date been found for three of the yeast mitochondrial ribosomal proteins only. Mrps17p and Mrpl37p may play a special role in yeast sporulation [33]. The mitochondrial ribosomal protein of the small subunit, yDAP-3 [34] is well conserved between yeast and human cells and besides its translational role has a distinct function in apoptosis. Its role in the aging process has not been studied yet.

**Figure 9.**
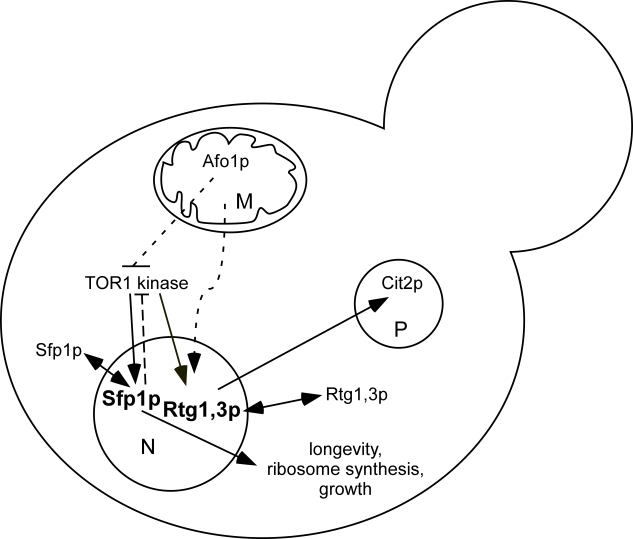
Schematic diagram of genetic interactions involving *AFO1 *based on the results presented in this paper. Dashed arrows: genetic interactions for which a molecular mechanism has not been determined. Both Sfp1p and Rtg1,3p shuttle to the cytoplasm when Tor1p is inhibited by rapamycin. They are indicated in bold in the nucleus, where they are active. An activating influence of the *TOR1 *kinase complex on the transcription factor *Rtg1*/*Rtg3 *has been postulated by Dann [5]. Feedback inhibition of Tor1p by nuclear Sfp1p is indicated. The RAS/cAMP and *SCH9 *components are omitted for clarity. Their interaction with the TOR pathway is complex. M, mitochondrion; N, nucleus; P, peroxisome.

Afo1p is a protein of *S. cerevisae *for which an obvious homolog is known in *Neurospora crassa *[16], but which has no easily apparent counterparts in other eukaryotes (or in *E. coli*) as judged by sequence similarity alone. It is therefore impossible presently to draw conclusions about possible functions of homologs of this protein in aging of higher eukaryotes. However, this may change when the three-dimensional structure of mitochondrial ribosomes will be determined and structural and functional homologs of Afo1p in higher eukaryotes may be found.

## Conclusion

In conclusion, we have shown that deletion of a gene coding for a mitochondrial ribosomal protein of yeast (*AFO1*, systematic name: YGR076C) leads not only to respiratory deficiency (as expected), but also to oxidative stress resistance, very low internal production of ROS and a substantial (60%) increase in the mother cell-specific lifespan of the strain. This was unexpected because a *bona fide *ρ^0^strain derived from the same parental yeast displayed no increase in lifespan. The lifespan effect of the mutant depends on the presence of a functional *TOR1 *gene. The relatively large effect on lifespan which *afo1Δ *confers is, however, independent of the presence or absence of ERCs in the aging mother cells. These experimental results show once again that replicative aging is multifactorial and that the limiting factor for the determination of the replicative lifespan may be very different for different strains and for different growth conditions. The physiological characterization of the long-lived mutant shows a relationship of the yeast replicative aging process to two cellular processes that have also been found to determine aging in higher organisms: i) nutritional signaling through the highly conserved TOR pathway, and ii) generation of and defense against internally generated oxidative stress molecules (ROS).

## Materials and methods

Media. The following media were used in this study: complex medium (YPD) containing 1% yeast extract, 2% (w/v) peptone and 2% (w/v) D-glucose; synthetic complete glucose medium (SC-glucose) containing 2% (w/v) D-glucose, 0.17% yeast nitrogen base without amino acids and ammonium sulphate, 0.5% ammonium sulphate and 10 mL complete dropout; synthetic complete raffinose (SC-raffinose), synthetic complete glycerol medium (SC-glycerol) or synthetic complete lactate medium (SC-lactate), containing the same ingredients as SC-glucose, except that 2% (w/v) D-glucose is replaced by 2% (w/v) raffinose, 2% (v/v) glycerol or 3% (w/v) lactate as a carbon source; synthetic minimal medium (SD) containing 2% (w/v) D-glucose, 0.17% yeast nitrogen base without amino acids and ammonium sulphate and 0.5% ammonium sulphate. Complete dropout contains: 0.2% Arg, 0.1%, His, 0.6% Ile, 0.6% Leu, 0.4% Lys, 0.1% Met, 0.6% Phe, 0.5% Thr, 0.4% Trp, 0.1% Ade, 0.4% Ura, 0.5% Tyr. Agar plates were made by adding 2% (w/v) agar to the media.

Strains. *S. cerevisiae *strains BY4741 and BY4742 (EUROSCARF) were used. For experiments with deletion strains we used the EUROSCARF deletion mutant collection (http://www.rz.unifrankfurt.de/FB/fb16/mikro/euroscarf/index.html). Other strains were obtained from the "Yeast-GFP clone collection" (Invitrogen Cooporation, Carlsbad, California,  USA) or the "TetO_7_promoter collection" (Open Biosystems, Huntsville, AL, USA). Double mutants were constructed by isogenic crossing of two single mutants of opposing mating type in the BY4741 background followed by sporulation of the obtained zygote and dissection of meiotic tetrads. A *bona fide *ρ^0^petites strain was made from the BY4741 wild type -strain as described in [35]. Briefly the strain was grown from a small inoculum to saturation in synthetic minimal medium (SD) containing the auxotrophic requirements plus 25μg/mL ethidium bromide. A second culture was inoculated from the first in the same medium and grown to saturation. This culture was streaked out for single colonies on YPD plates and checked for petite character by growth on YPG (complex medium containing 2% (v/v) glycerol as sole carbon source). To transfer the *SFP1*-*GFP*-*HIS3 *chromosomal integrated GFP construct into the *afo1Δ *and *bona fide *BY4741 ρ^0^strain, the *SFP1*-*GFP*-*HIS3* construct was PCR cloned and chromosomally integrated at the *SFP1 *locus of the *afo1*Δ and BY4741 ρ^0^strains, respectively.

Elutriation. Cells were separated according to their diameter using the Beckman elutriation system and rotor JE-6B with a standard elutriation chamber. Before the separation, the cells were grown in 100 mL of YPD medium at 28°C on a rotary shaker for 24 h. Then, the cells were harvested at 3000 rpm and resuspended in 1X PBS buffer (8 g of NaCl, 0.2 g of KCl, 1.44 g of Na_2_HPO_4_, 0.24 g of KH_2_PO_4_, pH 7.4, in a total volume of 1 L) at 4°C. The elutriation chamber was loaded with 4.2 mL of cell suspension corresponding to about 109 cells. To separate cell fractions with different diameters, the chamber was loaded at a flow rate of 10 mL/min and a rotor speed of 3200 rpm. Cells with a diameter <5 μm were elutriated (fraction I). To collect fraction II (diameter 5-7 μm), the flow rate was set to 15 mL/min and a rotor speed to 2700 rpm. Fraction III (diameter 7-8.5 μm) was elutriated at 2400 rpm., fraction IV (diameter 8.5-10 μm) at 2000 rpm. and, finally, fraction V (diameter 10-15 μm) at 1350 rpm. The quality of separation of particular fractions was verified microscopically. Note that in the separation of the slightly smaller *afo1Δ *mutant cells no significant  amount of fraction V cells could be isolated. Therefore, fraction IV was used for ERC determination.

Replicative lifespan. The replicative lifespan measure-ments were performed as described previously [2]. All lifespans were determined on defined SC-glucose media for a cohort of at least 45 cells. Standard deviations of the median lifespans were calculated according to Kaplan-Meier statistics [36]. Median lifespan is the best-suited single parameter to describe a lifespan distribution. To determine whether two given lifespan distributions are significantly different at the 98% confidence level, Breslow, Tarone-Ware and log-rank statistics were used. All statistical calculations were performed using the software package SPSS 15.0 (SPSS Inc., Chicago, IL, USA).

Sensitivity to oxidants. Plate tests for sensitivity to oxidants were performed by spotting cell cultures onto SC-glucose plates containing various concentrations of H_2_O_2_(2-4 mM) and t-BHP (0.8-2 mM). Cells were grown to stationary phase in liquid SC-glucose, serially diluted to OD_600_values of 3.0; 1.0; 0.3; 0.1 and 10 μL aliquots were spotted onto the appropriate plates. Sensitivity was determined by comparison of growth with that of the wild-type strain after incubation at 28°C for three days. RNA preparation and Northern analysis RNA was prepared from log-phase cells in SC-glucose and SC-raffinose with the RNeasy Midi Kit (Qiagen, Vienna, Austria). Heat-denatured RNA samples (10 μg) were separated by electrophoresis (5 h, 5 V/cm) in a 1.3% (w/v) agarose gel containing 0.6 M formaldehyde, transferred to a nylon membrane, and immobilized by irradiation with UV light (UV Stratalinker 1800, Stratagene, La Jolla, CA). Membranes were pre-hybridized for 2 h at 60°C in 10 mL Church Gilbert solution (0.5 M Na_2_HPO_4_, 1 mM EDTA, 7% (w/v) SDS, 1% BSA) and 100 μl (10 mg/mL stock solution) single-stranded denatured salmon sperm DNA, and then probed under the same conditions for 16 h with *CIT2* and *ACT1* probes which were labelled with ^32^P-dCTP by random oligonucleotide priming. After hybridisation, the filters were washed two times for 15 min with 2xSSC/0.1% SDS at room temperature, followed by two 15-min washes with 0.2xSSC/0.1% SDS at 56°C. Blots were wrapped in Saran Wrap and exposed for 15 min to an imaging cassette (Fujifilm BAS cassette 2325). Images were scanned in a Phosphoimager (Fujifilm BAS 1800II) using the BASreader 2.26 software.

Sfp1-GFP localization experiment. Strains of interest were grown overnight from a small inoculum to saturation in SC-glucose medium. These cultures were taken to inoculate 25 mL of SC-glucose medium in such a way that cultures were in the early exponential growth phase on the next morning. A sample was taken for confocal microscopy (cells without rapamycin treatment). The rest of the culture was treated for 4 hours with 100 nM rapamycin (LC Laboratories, Woburn, MA, USA) and further inoculated at 28°C. Before cells were used for confocal microscopy, cells were harvested by centrifugation and resuspended in fresh SC-glucose medium. For the arsenite inhibition experiments, the cells were grown in synthetic medium to an OD_600_of 1. As_2_O_3_was added to the cells to a final concentration of 0.5 mM. Live fluorescence pictures were taken after 10 minutes incubation at 30°C. Markers of apoptosis were determined as described in [2]. ERCs were determined as described in [19].

## Supplementary figures

Supplementary Figure 1 Northern blots (see also experimental procedures) of CIT2 showing the absence of the retrograde response [15] in the *afo1Δ* strain grown on 2% glucose.

Supplementary Figure 2 Subcellular localization of AFO1-GFP. Exponentially growing cells of strain YUG37 [38] transformed with the Afo1p-GFP construct in pMR2 under a tetracyclin- regulatable promoter were induced with doxycyclin, stained with Mitotracker deep red and analyzed with a Leica confocal microscope. (**A**) Cells stained with Mitotracker deep red; (**B**) the same cells as in (**A**) stained with Afo1p-GFP; (**C**) the same cells in phase contrast; (**D**) overlay of (**A**) and (**B**). (**E-H**) The same technique as in (**A**) to (**D**) was applied to a senescent mother cell (fraction V) of the same strain. (**I**) Strain JC 482 [39] transformed with plasmid pUG35 containing Afo1p-GFP under control of the MET25 promoter and grown to mid-log phase on SC-lactate was observed by confocal microscopy to reveal the mitochondrial localization of the protein.
