# Supplementary figures and images for: The mitochondrial ribosomal protein of the large subunit, Afo1p, determines cellular longevity through mitochondrial back-signaling via TOR1
Source: Aging (Albany NY). 2009 Jul 13;1(7):622–36. doi: 10.18632/aging.100065 (PMC2806038; doi:10.18632/aging.100065)

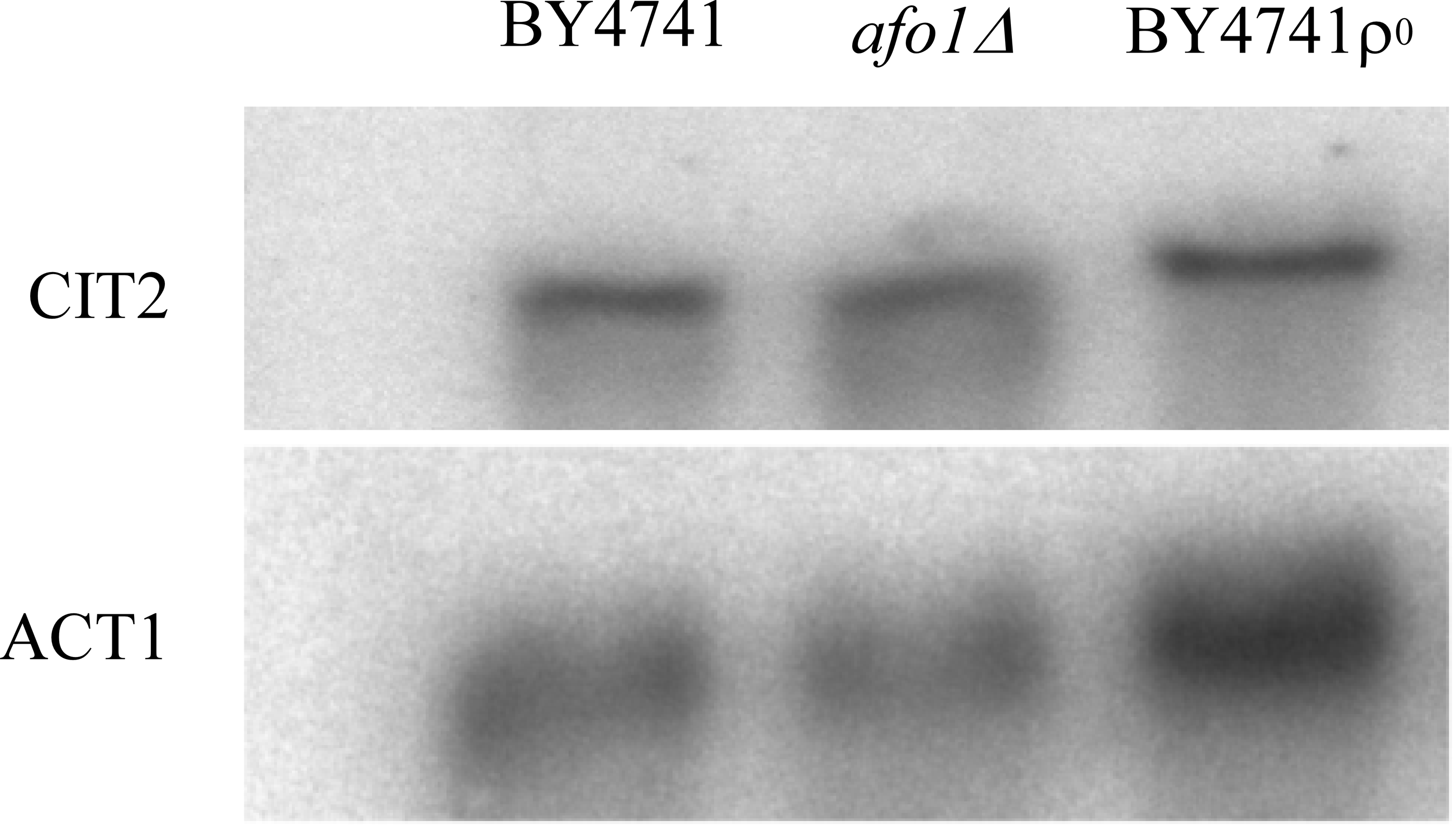

Supplement: Supplementary Figure 1 — Northern blots (see also experimental procedures) of CIT2 showing the absence of the retrograde response [15] in the afo1Δ strain grown on 2% glucose. [file aging-01-622-s001.tif]

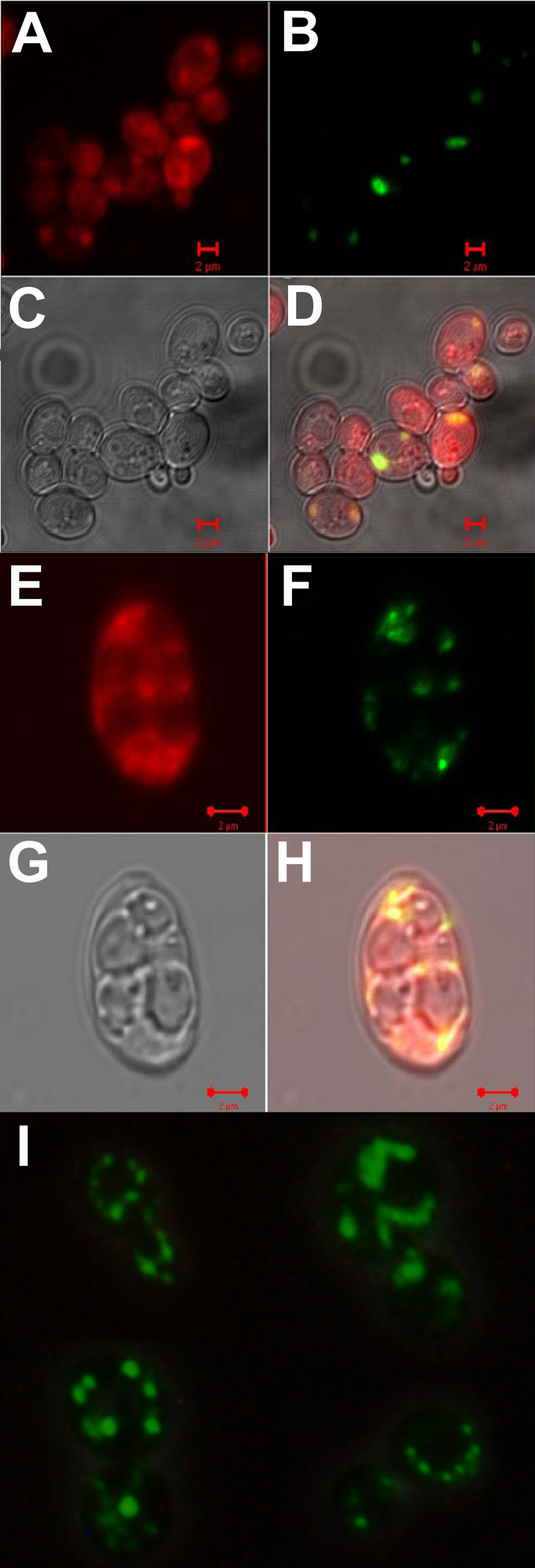

Supplement: Supplementary Figure 2 — Subcellular localization of AFO1-GFP. Exponentially growing cells of strain YUG37 [38] transformed with the Afo1p-GFP construct in pMR2 under a tetracyclin- regulatable promoter were induced with doxycyclin, stained with Mitotracker deep red and analyzed with a Leica confocal microscope. (A) Cells stained with Mitotracker deep red; (B) the same cells as in (A) stained with Afo1p-GFP; (C) the same cells in phase contrast; (D) overlay of (A) and (B). (E-H) The same technique as in (A) to (D) was applied to a senescent mother cell (fraction V) of the same strain. (I) Strain JC 482 [39] transformed with plasmid pUG35 containing Afo1p-GFP under control of the MET25 promoter and grown to mid-log phase on SC-lactate was observed by confocal microscopy to reveal the mitochondrial localization of the protein. [file aging-01-622-s002.tif]
